# Attitudes towards people who use substances: a survey of mental health clinicians from an urban hospital in British Columbia

**DOI:** 10.1186/s12954-023-00733-w

**Published:** 2023-01-19

**Authors:** Angela Russolillo, Meijiao Guan, Elizabeth J. Dogherty, Maja Kolar, Jennifer Du, Elísabet Brynjarsdóttir, Michelle Carter

**Affiliations:** 1 Mental Health Program, Providence Health Care, St. Paul’s Hospital, 1081 Burrard Street, Vancouver, BC V6Z 1Y6 Canada; 2 Centre for Health Evaluation and Outcome Sciences, 588-1081 Burrard Street, Vancouver, BC V6Z 1Y6 Canada; 3 Faculty of Health Sciences, Simon Fraser University, 8888 University Dr, Burnaby, BC V5A 1S6 Canada; 4 Providence Health Care, St. Paul’s Hospital, 1081 Burrard Street, Vancouver, BC V6Z 1Y6 Canada

## Abstract

Stigma and other barriers limit harm reduction practice integration by clinicians within acute psychiatric settings. The objective of our study was to explore mental health clinician attitudes towards substance use and associations with clinical experience and education level. The Brief Substance Abuse Attitudes Survey was completed among a convenience sample of mental health clinicians in Vancouver, British Columbia. Five predefined attitude subgroups were evaluated. Respondents’ attitudes towards substance use were associated with level of education on questions from two (non-stereotyping [*p* = 0.012] and treatment optimism [*p* = 0.008]) subscales. In pairwise comparisons, postgraduate education was associated with more positive attitudes towards relapse risk (*p* = 0.004) when compared to diploma-educated respondents. No significant associations were observed between years of clinical experience and participant responses. Our findings highlight important aspects of clinician attitudes that could improve harm reduction education and integration into clinical practice.

## Introduction

North America is currently in the midst of an unprecedented opioid epidemic. The number of opioid-related deaths is rising steadily across Canada, with the highest mortality rate occurring in British Columbia [1]. Many individuals with opioid use present with several comorbidities including co-occurring mental illness and polysubstance use [2]. These factors contribute to several cross-cutting health and social challenges, often limiting treatment initiation and increasing opioid-related harms [3]

Individuals with both psychiatric and substance use issues are at increased risk for drug-related death [4], especially following periods of abstinence and reduced tolerance, such as hospitalization [5]. Harm reduction interventions, such as take-home naloxone and supervised consumption sites, are important elements of care and are associated with a reduced risk of overdose death [6]. Despite a robust body of evidence supporting the public health benefit of harm reduction interventions [7], stigma and related barriers (e.g. discrimination, lack of knowledge) can limit their availability across the health system [8]. Existing research consistently reveals that health care professionals hold negative beliefs about people with substance use disorders [9], and these attitudes may worsen over time for dually diagnosed patients [10]. Unfortunately, these stigmatized perceptions, such as abuse of health system resources or failure to adhere to recommended care and treatment [11, 12], often contribute to inequitable and poor provision of care including reduced access to harm reduction resources [13]. For example, in acute psychiatric settings addiction-related stigma among clinicians has been associated with poor harm reduction integration [14, 15]. The acceptability of harm reduction approaches among clinicians is an important determinant of increased implementation of essential, evidence-based services to combat the ongoing opioid epidemic.

Given the disconnect between the scientific evidence for and the resistance to harm reduction among health care providers, it is critical to examine how clinician attitudes towards substance use influence the harm reduction best practices in psychiatric settings. The objective of our study was to explore mental health clinician attitudes towards substance use and associations with clinical experience and education level.

## Methods

We conducted an online cross-sectional survey among a convenience sample (*n* = 71) of mental health clinicians (nurses, physicians and allied health) between May and June 2022 at a tertiary care hospital in Vancouver, British Columbia. Participants were recruited via email based on internal distribution lists and informed consent was obtained electronically. Eligible participants were employed fulltime, parttime or casual within the hospital and had at least one year of experience in an acute psychiatric setting. The survey included socio-demographic questions (gender, education, clinical experience) and the 25-item Brief Substance Abuse Attitudes Survey (BSAAS). The BSAAS [16] was derived from a 50-item instrument, the Substance Abuse Attitudes Survey, using a Likert-type response format ranging from 1 (strongly disagree) to 5 (strongly agree) to examine attitudes towards various aspects of drug and alcohol use and five predefined attitude subgroups were evaluated: permissiveness, non-stereotypes, treatment intervention, treatment optimism and non-moralism [17]. Chi-square test or Fisher’s exact tests were used to examine the association between attitudes towards substance use and years of clinical experience and education level. Hochberg procedure [18] was applied to adjust *p* values for multiple comparisons. Statistical analyses were performed using SAS version 9.4. Ethics approval was obtained from the Providence Health Care/University of British Columbia Review Board.

## Results

Among the 71 respondents who completed the survey, the majority identified as female (85%), were employed as nurses (48%), with bachelor’s (44%) or master’s (23%) level education and fewer than ten years of experience in their primary clinical role (71%) (Table 1). Over half (60%) of respondents reported a high level of daily contact (> 71% of workload) with individuals who use substances, but low levels of comfort in providing care and treatment (Table 1).

**Table 1:** Socio-demographic characterisitcs of respondents

Gender	N(%)
Female	60 (85)
Male	9 (13)
Non-binary	1 (1)
Prefer not to answer	1 (1)
Highest level of education	
Diploma	15 (21)
Bachelor’s degree	31 (44)
Master’s degree	16 (23)
Doctorate degree	3 (4)
Prefer not to answer	4 (6)
Primary clinical role	
Physician	2 (3)
Nurse, Registered Psychiatric Nurse	29 (41)
Nurse, Registered Nurse	6 (8)
Allied Health (e.g., social worker, psychologist)	19 (27)
Other (e.g. manager, dietician, care aide)	15 (21)
Years of clinical experience in primary clinical role	
1–5	31 (44)
6–10	19 (27)
11–15	5 (7)
16–20	8 (11)
21–25	4 (6)
26–30	2 (3)
31+	2 (3)
Primary work setting	
Inpatient unit	46 (65)
Emergency department	4 (6)
Outpatient clinic	13 (18)
Other	8 (11)
Proportion (%) of daily clinical patient volume/workload includes individuals with substance use or dependence	
0–10	6 (8)
11–20	4 (6)
21–30	4 (6)
31–40	3 (4)
41–50	4 (6)
51–60	3 (4)
61–70	5 (7)
71–80	16 (23)
81–90	13 (18)
91–100	13 (18)
Comfortability providing care and treatment for individuals with substance use disorders	
Extremely comfortable	18 (25)
Somewhat comfortable	23 (32)
Neither comfortable nor uncomfortable	15 (21)
Somewhat uncomfortable	12 (17)
Extremely uncomfortable	3 (4)

Respondents’ attitudes and views towards substance use were associated with level of education on questions from two subgroups, non-stereotyping (*p* = 0.012) and treatment optimism (*p* = 0.008) (Table 2). In pairwise comparisons within the treatment optimism subgroup, postgraduate education was associated with more positive attitudes towards relapse risk (*p* = 0.004) when compared to diploma-educated respondents. In contrast, no pairwise effect was observed between education type with respect to propensity for hard drug use (*p* = 0.310) within the non-stereotyping subgroup. No significant associations were demonstrated between years of clinical experience and attitudes towards substance use (Table 2).

**Table 2:** Attitudes towards substance use and associations with years of clinical experience and level of education (n = 71)

Variables	All (N = 71)	1–5 y (N = 31)	6–10 y (N = 19)	> 10 y (N = 21)	*P* value	Adjusted *p* value	Missing
Permissive category	n (%)						
Q3.1.7 Daily use of marijuana is not necessarily harmful					0.496		0
Strongly agree	8 (11.3)	4 (12.9)	3 (15.8)	1 (4.8)			
Somewhat agree	27 (38.0)	13 (41.9)	8 (42.1)	6 (28.6)			
Undecided	8 (11.3)	2 (6.5)	2 (10.5)	4 (19)			
Somewhat disagree	20 (28.2)	10 (32.3)	5 (26.3)	5 (23.8)			
Strongly disagree	8 (11.3)	2 (6.5)	1 (5.3)	5 (23.8)			
Q3.2.4 Lifelong abstinence is a necessary goal in the treatment of substance use					0.549		0
Strongly agree	7 (9.9)	2 (6.5)	1 (5.3)	4 (19)			
Somewhat agree	24 (33.8)	9 (29)	7 (36.8)	8 (38.1)			
Undecided	4 (5.6)	1 (3.2)	2 (10.5)	1 (4.8)			
Somewhat disagree	17 (23.9)	7 (22.6)	5 (26.3)	5 (23.8)			
Strongly disagree	19 (26.8)	12 (38.7)	4 (21.1)	3 (14.3)			
Q3.2.5 Once a person becomes drug free through treatment, they can never become a social user					0.241		0
Strongly agree	10 (14.1)	1 (3.2)	3 (15.8)	6 (28.6)			
Somewhat agree	8 (11.3)	4 (12.9)	3 (15.8)	1 (4.8)			
Undecided	16 (22.5)	8 (25.8)	4 (21.1)	4 (19)			
Somewhat disagree	26 (36.6)	11 (35.5)	6 (31.6)	9 (42.9)			
Strongly disagree	11 (15.5)	7 (22.6)	3 (15.8)	1 (4.8)			
Non-stereotype category							
Q3.1.5 Smoking leads to marijuana use, which in turn leads to hard drugs					0.785		0
Strongly agree	1 (1.4)	0 (0.0)	1 (5.3)	0 (0.0)			
Somewhat agree	1 (1.4)	1 (3.2)	0 (0.0)	0 (0.0)			
Undecided	1 (1.4)	0 (0.0)	0 (0.0)	1 (4.8)			
Somewhat disagree	12 (16.9)	6 (19.4)	3 (15.8)	3 (14.3)			
Strongly disagree	56 (78.9)	24 (77.4)	15 (78.9)	17 (81)			
Q3.2.8 A hospital is the best place to treat an individual with substance use issues					0.498		0
Strongly agree	2 (2.8)	1 (3.2)	0 (0.0)	1 (4.8)			
Somewhat agree	8 (11.3)	5 (16.1)	3 (15.8)	0 (0.0)			
Undecided	11 (15.5)	5 (16.1)	4 (21.1)	2 (9.5)			
Somewhat disagree	33 (46.5)	13 (41.9)	9 (47.4)	11 (52.4)			
Strongly disagree	17 (23.9)	7 (22.6)	3 (15.8)	7 (33.3)			
Non-moralism category							
Q3.1.9 A physician who has been addicted to narcotics should not be allowed to practise medicine again					0.274		0
Strongly agree	1 (1.4)	0 (0.0)	0 (0.0)	1 (4.8)			
Somewhat agree	2 (2.8)	0 (0.0)	2 (10.5)	0 (0.0)			
Undecided	12 (16.9)	6 (19.4)	3 (15.8)	3 (14.3)			
Somewhat disagree	25 (35.2)	14 (45.2)	6 (31.6)	5 (23.8)			
Strongly disagree	31 (43.7)	11 (35.5)	8 (42.1)	12 (57.1)			
Q3.1.1 Alcoholism is associated with a weak will					0.625		0
Somewhat agree	4 (5.6)	3 (9.7)	0 (0.0)	1 (4.8)			
Somewhat disagree	13 (18.3)	4 (12.9)	4 (21.1)	5 (23.8)			
Strongly disagree	54 (76.1)	24 (77.4)	15 (78.9)	15 (71.4)			
Q3.3.5 A nurse with a substance use disorder should not be allowed to give medications to patients					0.524		1
Strongly agree	2 (2.9)	1 (3.3)	0 (0.0)	1 (4.8)			
Somewhat agree	5 (7.1)	2 (6.7)	1 (5.3)	2 (9.5)			
Undecided	14 (20.0)	8 (26.7)	3 (15.8)	3 (14.3)			
Somewhat disagree	20 (28.6)	10 (33.3)	7 (36.8)	3 (14.3)			
Strongly disagree	29 (41.4)	9 (30.0)	8 (42.1)	12 (57.1)			
Treatment intervention category							
Q3.1.6 Physicians who diagnose alcoholism early improve the chance of treatment success					0.357		0
Strongly agree	13 (18.3)	3 (9.7)	3 (15.8)	7 (33.3)			
Somewhat agree	32 (45.1)	15 (48.4)	11 (57.9)	6 (28.6)			
Undecided	17 (23.9)	9 (29)	4 (21.1)	4 (19)			
Somewhat disagree	6 (8.5)	2 (6.5)	1 (5.3)	3 (14.3)			
Strongly disagree	3 (4.2)	2 (6.5)	0 (0.0)	1 (4.8)			
Q3.2.7 Group therapy is very important in the treatment of substance use					0.912		0
Strongly agree	32 (45.1)	13 (41.9)	9 (47.4)	10 (47.6)			
Somewhat agree	23 (32.4)	11 (35.5)	6 (31.6)	6 (28.6)			
Undecided	14 (19.7)	7 (22.6)	3 (15.8)	4 (19)			
Somewhat disagree	2 (2.8)	0 (0.0)	1 (5.3)	1 (4.8)			
Q3.1.8 Urine drug screening can be an important part of substance use treatment					0.701		0
Strongly agree	15 (21.1)	6 (19.4)	3 (15.8)	6 (28.6)			
Somewhat agree	35 (49.3)	14 (45.2)	11 (57.9)	10 (47.6)			
Undecided	12 (16.9)	4 (12.9)	4 (21.1)	4 (19)			
Somewhat disagree	8 (11.3)	6 (19.4)	1 (5.3)	1 (4.8)			
Strongly disagree	1 (1.4)	1 (3.2)	0 (0.0)	0 (0.0)			
Treatment optimism category							
Q3.2.6 Substance use is a treatable illness					0.436		0
Strongly agree	45 (63.4)	17 (54.8)	12 (63.2)	16 (76.2)			
Somewhat agree	23 (32.4)	12 (38.7)	7 (36.8)	4 (19)			
Undecided	3 (4.2)	2 (6.5)	0 (0.0)	1 (4.8)			
Q3.2.9 Alcoholism is a treatable illness					0.493		0
Strongly agree	47 (66.2)	19 (61.3)	11 (57.9)	17 (81)			
Somewhat agree	21 (29.6)	10 (32.3)	7 (36.8)	4 (19)			
Undecided	1 (1.4)	1 (3.2)	0 (0.0)	0 (0.0)			
Somewhat disagree	1 (1.4)	0 (0.0)	1 (5.3)	0 (0.0)			
Strongly disagree	1 (1.4)	1 (3.2)	0 (0.0)	0 (0.0)			
Q3.2.1 An individual who uses alcohol or drugs and has relapsed several times probably cannot be treated					0.86		0
Undecided	2 (2.8)	1 (3.2)	0 (0.0)	1 (4.8)			
Somewhat disagree	19 (26.8)	9 (29)	6 (31.6)	4 (19)			
Strongly disagree	50 (70.4)	21 (67.7)	13 (68.4)	16 (76.2)			
Q3.1.2 An individual who uses alcohol or drugs cannot be helped until they hit ‘rock bottom’					0.675		0
Strongly agree	1 (1.4)	0 (0.0)	0 (0.0)	1 (4.8)			
Somewhat agree	3 (4.2)	0 (0.0)	2 (10.5)	1 (4.8)			
Undecided	5 (7.0)	2 (6.5)	2 (10.5)	1 (4.8)			
Somewhat disagree	17 (23.9)	8 (25.8)	4 (21.1)	5 (23.8)			
Strongly disagree	45 (63.4)	21 (67.7)	11 (57.9)	13 (61.9)			
Q3.3.1 Most individuals who use alcohol or drugs are unpleasant to work with as patients					0.541		1
Somewhat agree	7 (10.0)	1 (3.3)	2 (10.5)	4 (19)			
Undecided	3 (4.3)	2 (6.7)	1 (5.3)	0 (0.0)			
Somewhat disagree	22 (31.4)	11 (36.7)	6 (31.6)	5 (23.8)			
Strongly disagree	38 (54.3)	16 (53.3)	10 (52.6)	12 (57.1)			

Variables	All (N = 67)	Diploma (N = 15)	Bachelor’s degree (N = 31)	Postgraduate (Master’s, PhD, MD) ( N = 21)	*P* value	Adjusted *p* value	Missing
Permissive category							
Q3.1.7 Daily use of marijuana is not necessarily harmful					0.248		0
Strongly agree	6 (9.0)	0 (0.0)	4 (12.9)	2 (9.5)			
Somewhat agree	26 (38.8)	3 (20.0)	15 (48.4)	8 (38.1)			
Undecided	8 (11.9)	4 (26.7)	3 (9.7)	1 (4.8)			
Somewhat disagree	19 (28.4)	6 (40.0)	7 (22.6)	6 (28.6)			
Strongly disagree	8 (11.9)	2 (13.3)	2 (6.5)	4 (19)			
Q3.2.4 Lifelong abstinence is a necessary goal in the treatment of substance use					0.071		0
Strongly agree	6 (9.0)	3 (20.0)	1 (3.2)	2 (9.5)			
Somewhat agree	22 (32.8)	6 (40.0)	13 (41.9)	3 (14.3)			
Undecided	4 (6.0)	2 (13.3)	2 (6.5)	0 (0.0)			
Somewhat disagree	17 (25.4)	2 (13.3)	8 (25.8)	7 (33.3)			
Strongly disagree	18 (26.9)	2 (13.3)	7 (22.6)	9 (42.9)			
Q3.2.5 Once a person becomes drug free through treatment, they can never become a social user					0.094		0
Strongly agree	10 (14.9)	3 (20.0)	4 (12.9)	3 (14.3)			
Somewhat agree	7 (10.4)	4 (26.7)	3 (9.7)	0 (0.0)			
Undecided	14 (20.9)	4 (26.7)	8 (25.8)	2 (9.5)			
Somewhat disagree	26 (38.8)	4 (26.7)	11 (35.5)	11 (52.4)			
Strongly disagree	10 (14.9)	0 (0.0)	5 (16.1)	5 (23.8)			
Non-stereotype category							
Q3.1.5 Smoking leads to marijuana use, which in turn leads to hard drugs					0.012		0
Strongly agree	1 (1.5)	0 (0.0)	1 (3.2)	0 (0.0)			
Somewhat agree	1 (1.5)	1 (6.7)	0 (0.0)	0 (0.0)			
Undecided	1 (1.5)	1 (6.7)	0 (0.0)	0 (0.0)			
Somewhat disagree	12 (17.9)	1 (6.7)	10 (32.3)	1 (4.8)			
Strongly disagree	52 (77.6)	12 (80.0)	20 (64.5)	20 (95.2)			
Pairwise comparison							
Postgraduate versus bachelor’s						0.072	
Diploma versus bachelor’s						0.094	
Postgraduate versus diploma						0.310	
Q3.2.8 A hospital is the best place to treat an individual with substance use issues					0.494		0
Strongly agree	1 (1.5)	0 (0.0)	1 (3.2)	0 (0.0)			
Somewhat agree	8 (11.9)	3 (20.0)	3 (9.7)	2 (9.5)			
Undecided	11 (16.4)	5 (33.3)	4 (12.9)	2 (9.5)			
Somewhat disagree	31 (46.3)	5 (33.3)	14 (45.2)	12 (57.1)			
Strongly disagree	16 (23.9)	2 (13.3)	9 (29)	5 (23.8)			
Non-moralism category							
Q3.1.9 A physician who has been addicted to narcotics should not be allowed to practise medicine again					0.983		0
Strongly agree	1 (1.5)	0 (0.0)	1 (3.2)	0 (0.0)			
Somewhat agree	2 (3.0)	0 (0.0)	1 (3.2)	1 (4.8)			
Undecided	8 (11.9)	2 (13.3)	4 (12.9)	2 (9.5)			
Somewhat disagree	25 (37.3)	5 (33.3)	13 (41.9)	7 (33.3)			
Strongly disagree	31 (46.3)	8 (53.3)	12 (38.7)	11 (52.4)			
Q3.1.1 Alcoholism is associated with a weak will					0.544		0
Somewhat agree	4 (6.0)	2 (13.3)	2 (6.5)	0 (0.0)			
Somewhat disagree	13 (19.4)	3 (20.0)	5 (16.1)	5 (23.8)			
Strongly disagree	50 (74.6)	10 (66.7)	24 (77.4)	16 (76.2)			
Q3.3.5 A nurse with a substance use disorder should not be allowed to give medications to patients					0.87		1
Strongly agree	2 (3.0)	0 (0.0)	1 (3.3)	1 (4.8)			
Somewhat agree	5 (7.6)	0 (0.0)	3 (10.0)	2 (9.5)			
Undecided	12 (18.2)	4 (26.7)	6 (20.0)	2 (9.5)			
Somewhat disagree	19 (28.8)	5 (33.3)	7 (23.3)	7 (33.3)			
Strongly disagree	28 (42.4)	6 (40.0)	13 (43.3)	9 (42.9)			
Treatment intervention category							
Q3.1.6 Physicians who diagnose alcoholism early improve the chance of treatment success					0.953		0
Strongly agree	13 (19.4)	2 (13.3)	6 (19.4)	5 (23.8)			
Somewhat agree	30 (44.8)	7 (46.7)	15 (48.4)	8 (38.1)			
Undecided	16 (23.9)	4 (26.7)	6 (19.4)	6 (28.6)			
Somewhat disagree	5 (7.5)	1 (6.7)	2 (6.5)	2 (9.5)			
Strongly disagree	3 (4.5)	1 (6.7)	2 (6.5)	0 (0.0)			
Q3.2.7 Group therapy is very important in the treatment of substance use					0.192		0
Strongly agree	31 (46.3)	9 (60.0)	16 (51.6)	6 (28.6)			
Somewhat agree	22 (32.8)	2 (13.3)	10 (32.3)	10 (47.6)			
Undecided	12 (17.9)	3 (20.0)	5 (16.1)	4 (19)			
Somewhat disagree	2 (3.0)	1 (6.7)	0 (0.0)	1 (4.8)			
Q3.1.8 Urine drug screening can be an important part of substance use treatment					0.457		0
Strongly agree	13 (19.4)	1 (6.7)	8 (25.8)	4 (19)			
Somewhat agree	34 (50.7)	7 (46.7)	17 (54.8)	10 (47.6)			
Undecided	11 (16.4)	3 (20.0)	3 (9.7)	5 (23.8)			
Somewhat disagree	8 (11.9)	3 (20.0)	3 (9.7)	2 (9.5)			
Strongly disagree	1 (1.5)	1 (6.7)	0 (0.0)	0 (0.0)			
Treatment optimism category							
Q3.2.6 Substance use is a treatable illness					0.261		0
Strongly agree	43 (64.2)	11 (73.3)	18 (58.1)	14 (66.7)			
Somewhat agree	22 (32.8)	4 (26.7)	13 (41.9)	5 (23.8)			
Undecided	2 (3.0)	0 (0.0)	0 (0.0)	2 (9.5)			
Q3.2.9 Alcoholism is a treatable illness					0.617		0
Strongly agree	45 (67.2)	11 (73.3)	20 (64.5)	14 (66.7)			
Somewhat agree	19 (28.4)	3 (20.0)	10 (32.3)	6 (28.6)			
Undecided	1 (1.5)	0 (0.0)	0 (0.0)	1 (4.8)			
Somewhat disagree	1 (1.5)	1 (6.7)	0 (0.0)	0 (0.0)			
Strongly disagree	1 (1.5)	0 (0.0)	1 (3.2)	0 (0.0)			
Q3.2.1 An individual who uses alcohol or drugs and has relapsed several times probably cannot be treated					0.008		0
Undecided	2 (3.0)	0 (0.0)	1 (3.2)	1 (4.8)			
Somewhat disagree	17 (25.4)	8 (53.3)	8 (25.8)	1 (4.8)			
Strongly disagree	48 (71.6)	7 (46.7)	22 (71)	19 (90.5)			
Pairwise comparison							
Postgraduate versus diploma						0.004	
Postgraduate versus bachelor’s						0.149	
Diploma versus bachelor’s						0.172	
Q3.1.2 An individual who uses alcohol or drugs cannot be helped until they hit ‘rock bottom’					0.646		0
Somewhat agree	3 (4.5)	1 (6.7)	2 (6.5)	0 (0.0)			
Undecided	5 (7.5)	1 (6.7)	2 (6.5)	2 (9.5)			
Somewhat disagree	17 (25.4)	5 (33.3)	9 (29)	3 (14.3)			
Strongly disagree	42 (62.7)	8 (53.3)	18 (58.1)	16 (76.2)			
Q3.3.1 Most individual who use alcohol or drugs are unpleasant to work with as patients					0.453		1
Somewhat agree	7 (10.6)	3 (20.0)	1 (3.3)	3 (14.3)			
Undecided	3 (4.5)	1 (6.7)	2 (6.7)	0 (0.0)			
Somewhat disagree	20 (30.3)	5 (33.3)	9 (30.0)	6 (28.6)			
Strongly disagree	36 (54.5)	6 (40.0)	18 (60.0)	12 (57.1)			

## Discussion

We found that postgraduate education contributes to less stereotyping attitudes and a higher level of treatment optimism when working with individuals with substance use or dependence. These findings highlight important dimensions of clinician attitudes (e.g. beliefs) that could improve harm reduction education and integration into clinical practice.

Prior research has identified that negative attitudes are consistently viewed as barriers to evidence-based harm reduction practices [19]. Even though combating stigma has been a focus of public health professionals, there are few interventions that demonstrate measurable improvements towards stigmatizing beliefs among health care providers [20]. Livingston and colleagues [21] in a systematic review identified that on-the-job education, which includes contact-based training, has demonstrated improvements in reducing stigma but the evidence base remains equivocal. Our results add to the literature by demonstrating a significant effect between less stigmatizing views and individuals with postgraduate education. This finding is important and may reflect the focus on anti-discriminatory and anti-oppressive practices within postgraduate pedagogy which challenges personal values and social norms regarding substance use [22].

We observed no associations between years of experience and clinician attitudes in our sample. This nonsignificant finding was in contrast to our hypothesis and existing literature which suggests that more experience and therefore increased contact with individuals who use substances fosters more positive attitudes [23]; however, this effect appears to be stronger for the general public [24] than health care providers. Moreover, this null finding highlights opportunities to provide workplace education for clinicians across all stages of their career, regardless of experience level.

Further research is required to understand elements of education and training that are associated with more positive attitudes towards and improved care for individuals who use substances. The limitations of this study include a low proportion of non-nursing providers, generalizability to other regions or settings and the cross-sectional survey design with assessments of attitudes at a single time-point.

## Data Availability

The datasets used and/or analysed during the current study are available from the corresponding author on reasonable request.
